# Biomarker identification by interpretable maximum mean discrepancy

**DOI:** 10.1093/bioinformatics/btae251

**Published:** 2024-06-28

**Authors:** Michael F Adamer, Sarah C Brüningk, Dexiong Chen, Karsten Borgwardt

**Affiliations:** Department of Biosystems Science and Engineering, ETH Zurich, Basel 4056, Switzerland; Swiss Institute for Bioinformatics (SIB), Amphipôle, Quartier UNIL-Sorge, Lausanne 1015, Switzerland; Department of Biosystems Science and Engineering, ETH Zurich, Basel 4056, Switzerland; Swiss Institute for Bioinformatics (SIB), Amphipôle, Quartier UNIL-Sorge, Lausanne 1015, Switzerland; Department of Health Sciences and Technology, ETH Zurich, Zurich 8008, Switzerland; Department of Biosystems Science and Engineering, ETH Zurich, Basel 4056, Switzerland; Swiss Institute for Bioinformatics (SIB), Amphipôle, Quartier UNIL-Sorge, Lausanne 1015, Switzerland; Department of Machine Learning and Systems Biology, Max Planck Institute of Biochemistry, Martinsried 82152, Germany; Department of Biosystems Science and Engineering, ETH Zurich, Basel 4056, Switzerland; Swiss Institute for Bioinformatics (SIB), Amphipôle, Quartier UNIL-Sorge, Lausanne 1015, Switzerland; Department of Machine Learning and Systems Biology, Max Planck Institute of Biochemistry, Martinsried 82152, Germany

## Abstract

**Motivation:**

In many biomedical applications, we are confronted with paired groups of samples, such as treated versus control. The aim is to detect discriminating features, i.e. biomarkers, based on high-dimensional (omics-) data. This problem can be phrased more generally as a two-sample problem requiring statistical significance testing to establish *differences*, and *interpretations* to identify distinguishing features. The multivariate maximum mean discrepancy (MMD) test quantifies group-level differences, whereas statistically significantly associated features are usually found by univariate feature selection. Currently, few general-purpose methods simultaneously perform multivariate feature selection and two-sample testing.

**Results:**

We introduce a sparse, interpretable, and optimized MMD test (SpInOpt-MMD) that enables two-sample testing and feature selection in the same experiment. SpInOpt-MMD is a versatile method and we demonstrate its application to a variety of synthetic and real-world data types including images, gene expression measurements, and text data. SpInOpt-MMD is effective in identifying relevant features in small sample sizes and outperforms other feature selection methods such as SHapley Additive exPlanations and univariate association analysis in several experiments.

**Availability and implementation:**

The code and links to our public data are available at https://github.com/BorgwardtLab/spinoptmmd.

## 1 Introduction

A common data mining step in biological high-throughput experiments is to determine whether two sets of measurements differ and, if so, why (Landgrebe *et al.* 2004, Borgwardt *et al.* 2006, Fox and Dimmic 2006, Stegle *et al.* 2010). Key examples include differential gene expression analysis, determination of relevant single nucleotide polymorphisms in healthy and diseased patients, masking tumor regions in cancer patients for healthy controls, and many more. In this article, we introduce a novel method to not only determine the distributional differences but also show how to find “significant features” that translate to differentially expressed genes in expression data or identifying disease-related regions in images.

The statistical frameworks are two-sample testing and feature selection. Two-sample testing determines whether two groups are statistically different, whereas, in feature selection identification, the identification of the most distinguishing features between two groups is key for feature selection. Therefore, two-sample testing can be considered a global view of a grouped dataset whereas feature selection represents a local view. Many common feature selection methods use univariate tests (e.g. *t*-tests) (Schena *et al.* 1995) considered two-sample tests in a one-dimensional dataset. While univariate tests are easy to implement and intuitive given relative feature importance, they consider a single feature at a time. The multiple testing problem (Benjamini and Hochberg 1995) is of great importance here. Univariate tests do not take feature interaction into account and pooling *P*-values to perform a global two-sample test can be challenging. These issues are partly mitigated by, e.g. multivariate approaches via classification that compute a *P*-value on the output metrics (Lopez-Paz and Oquab 2016) to perform a two-sample test. The choice of classification model is, however, crucial to achieving sufficient test power. Furthermore, there is often no general way to rigorously perform feature selection with non-linear classifiers. A natural multivariate and non-linear approach is the kernel-based maximum mean discrepancy (MMD) two-sample test (Gretton *et al.* 2006) that has been widely used (Borgwardt *et al.* 2006, Gretton *et al.* 2012a). While MMD-based tests empirically often show high statistical power, they are limited regarding interpretation of the test results, i.e. MMD tests excel in two-sample testing, but offer no option to simultaneously perform feature selection. Recently, an interpretable kernel-based two-sample test was introduced by Jitkrittum *et al.* (2016). Here, a “test location” considered as a data point in the input space was fitted on the training set. While this test has superior test power *and* the test location informs on the distributional differences, the underlying optimization problem is highly non-convex. As such, the locus of the optimal test location can vary considerably and it needs to be averaged across many trials to be informative. Since the (averaged) test location is simply a point in the input space, the direct interpretation of this test location as a means of feature selection is not straightforward. In some benchmark datasets (NeurIPS) of Jitkrittum *et al.* (2016), features vary between 0 and 1 and, therefore, Jittkrittum et al. vaguely interpret the test location as “if its value for a certain feature is closer to 1 than to 0, we expect this feature to be relevant.” However, in scenarios other than benchmark datasets, where the feature distribution and range are more complex, it is not so clear-cut what the significance of the test location may be.

The overarching contribution of this method is to close the gap between a global two-sample test and local feature selection. We present SpInOpt-MMD, a **sp**arse, **in**terpretable, and **opt**imized MMD-based two-sample test, that has superior test power to the classical MMD approaches while also identifying distinguishing features. It provides a highly sensitive two-sample test and directly optimizes feature weights to obtain directly interpretable outputs rather than a test location. In the Section Methods, we formally introduce two-sample testing and describe our method. In the Section Experimental Setup, we describe the hyperparameter optimization procedures, and the baselines and introduce our datasets. In the Section Experimental Results we describe the datasets and interpret our results. In Conclusion, we summarize our findings and discuss further research directions.

## 2 Materials and methods

In this section, we briefly review the previous MMD tests (Gretton *et al.* 2012a, Jitkrittum *et al.* 2016) on which we build our approach. We then introduce SpInOpt-MMD and discuss its properties.

### 2.1 Maximum mean discrepancy tests

The problem of two-sample testing can be formally described as follows.

Definition 1. (Two-sample testing).

Let *P* and *Q* be two probability distributions on a topological space X. Further, let $X=\{x_{1},\ldots ,x_{m}\}$ and $Y=\{y_{1},\ldots ,y_{n}\}$ be independently and identically distributed (i.i.d.) samples from *P* and *Q*, respectively. A two-sample test gives a statistical estimate of whether P≠Q given the samples *X* and *Y.*

To address the two-sample testing problem, (Gretton *et al.* 2006) introduced the MMD.

Definition 2. Given a function class F and two samples *X* and *Y* from probability distributions *P* and *Q*, respectively, let the MMD be

(1) $$\text{MMD}[F,P,Q]:=\underset{f\in F}{\sup}\left(E_{X}[f(X)]\;-\;E_{Y}[f(Y)]\right),$$

where E[·] denotes the expectation value.

When the chosen function class F is a reproducing kernel Hilbert space (RKHS), then, the MMD is equivalent to the difference of the sample means in the embedding space of the kernel (Gretton *et al.* 2012a). In particular, for *characteristic kernels* (Fukumizu *et al.* 2007) the MMD is a metric on distributions on the space X, i.e. MMD[F,P,Q]=0 if and only if P=Q.

Several biased and unbiased estimators for the MMD can be constructed, most prominent ones are the unbiased quadratic-time estimator (MMD-quad) [Gretton *et al.* 2012a, Equation (3)] and the linear-time (MMD-lin) estimator (Gretton *et al.* 2012a, Lemma 14). Due to the superior performance of MMD-quad, we focus on this estimator in this study, however, all of our results can be applied to MMD-lin mutatis mutandis. The MMD-quad for samples of size *m* and *n*, respectively, is given by,

(2) $$\begin{array}{c} {\text{MMD}}_{\text{quad}}^{2}[F,X,Y]=\frac{1}{m(m\;-\;1)}\sum_{i=1}^{m}\sum_{j\neq i}^{m}k(x_{i},x_{j})\\ +\frac{1}{n(n\;-\;1)}\sum_{i=1}^{n}\sum_{j\neq i}^{n}k(y_{i},y_{j})\\ -\frac{2}{mn}\sum_{i=1}^{m}\sum_{j=1}^{n}k(x_{i},y_{j}), \end{array}$$

for some kernel k(·,·). The quadratic time complexity arises due to the double sum over the data points. The null distribution (i.e. X=Y) is dependent on the distribution *P* (=Q) and, therefore, it is usually approximated by permutations. The test power of MMD-quad can be approximated by $\text{Power}={\text{MMD}}_{\text{quad}}^{2}/V[{\text{MMD}}_{\text{quad}}^{2}]$ (Sutherland *et al.* 2017), where $V[{\text{MMD}}_{\text{quad}}^{2}]$ is a variance estimator of ${\text{MMD}}_{\text{quad}}^{2}$ (Liu *et al.* 2020). The remainder of this article is dedicated to improving this test power by using feature weights and a sparsity-inducing regularization.

### 2.2 SpInOpt: sparse, interpretable, optimized MMD tests

We consider a class of parameterized kernels that can be optimized to maximize the test power while retaining a minimal number of parameters. First, the MMD is rendered a metric by specifying a suitable kernel. As shown in (Fukumizu *et al.* 2007), most common kernels such as the radial basis function (RBF) kernel, the linear kernel, and polynomial kernels all have this property. We focus on the RBF kernel in this article due to its widespread use, but all our results also apply to the other kernels mentioned.

### 2.3 Most implementations of the RBF kernel follow the form


(3) $$k(x,y)=e^{-\frac{\sum_{i}{\left(x_{i}-y_{i}\right)}^{2}}{\sigma^{2}}}$$

for two samples $x,y\in R^{d}$ and a kernel bandwidth σ. If all features are on the same scale, then, Equation (3) gives equal weight to every feature in the dataset. However, in many high-dimensional distributions, it is only a small fraction of the dimensions (features) that vary between the distributions. Therefore, an improvement in test power can be made by only including features that increase the test power and remove unimportant features. It is usually computationally intractable to find such a feature set by brute force searching all $2^{d}$ combinations of features. Therefore, we relax the hard inclusion criterion by assigning a weight $w\in R^{d}$ to the features and obtain a rescaling $\tilde{x}=w⊙x$, where ⊙ denotes the Hadamard product. We then optimize these weights on a training partition of the data with the $\ell_{1}$-regularized test-power objective, given by

(4) $$\underset{w}{\max}\;\;\ell (X,Y;w)=\underset{w}{\max}\;\;\frac{{\text{MMD}}_{\text{quad}}^{2}(w⊙X,w⊙Y)}{V[{\text{MMD}}_{\text{quad}}^{2}(w⊙X,w⊙Y)]}-\frac{\lambda }{d}\ell_{1}(w),$$

where we use the shorthand $w⊙X=\{w⊙x_{1},\ldots ,w⊙x_{m}\}$ and $w⊙Y=\{w⊙y_{1},\ldots ,w⊙y_{n}\}$. The term $\ell_{1}(w)$ is a sparsity-inducing regularization, in our case $\ell_{1}(w)=\sum_{i=1}^{d}|w_{i}|$ and λ>0 is a regularization parameter. Optimizing Equation (4) serves two purposes; (i) it increases the test power and (ii) it gives the largest weights to features that differ most between the samples. We call the resulting two-sample test with optimized test power and sparse feature weights *SpInOpt-MMD*.

We first notice that the optimization objective of Equation (4) is not, in general, convex. And in particular, the RBF, linear, and polynomial kernels are invariant under the transformation $w_{i}\to -w_{i}\;\forall i\in \{1,\ldots ,d\}$. Therefore, without loss of generality, we only focus on *non-negative weights*. Although, we perform an unconstrained optimization of (4), we can always project the final solution to the non-negative orthant. The fitted weights are then used to perform an MMD test on the test partition of the data. As will be shown in Section Experimental Results this allows MMD-quad to distinguish between complex biological distributions.

The runtime complexity of optimizing Equation (4) depends on the optimization algorithm chosen. In particular, it depends on the number of MMD-quad computations and, therefore, on the convergence rate of the optimization algorithm. For a benchmark on test time, from which runtimes can be extrapolated, we refer to the Supplementary Section C.

Note that computing the SpInOpt-MMD is equivalent to using unweighted features and defining an RBF kernel

(5) $$k(x,y)=e^{-{\left(x\;-\;y\right)}^{T}\Sigma^{-1}(x\;-\;y)},$$

with inverse covariance matrix $\Sigma^{-1}=\text{diag}(w_{1}^{2},\ldots ,\ldots w_{d}^{2})/\sigma^{2}$ and diag(·) denoting a diagonal matrix. Therefore, optimizing Equation (4) corresponds to solving a kernel selection problem with sparse inverse covariance. This reformulation of our objective ties our results to the approach of (Sutherland *et al.* 2017) which discussed the use of kernel optimization in the context of generative adversarial neural networks. As a corollary, we are also guaranteed to always obtain a symmetric and positive definite kernel.

When the kernel in the SpInOpt-MMD estimator is a linear kernel, i.e. k(x,y)=x·y, our approach is closely related to the method of Gretton *et al.* (2012b). In Gretton *et al.* (2012b), the authors construct convex linear combinations of base kernels with a sum constraint on the linear coefficients, i.e. $\sum_{i=1}^{d}\beta_{i}=D$. Since they assume $\beta_{i}\geq 0\;\forall i\in \{1,\ldots ,d\}$, the sum constraint is equivalent to $\ell_{1}$ regularization. In the case of each base kernel being a linear kernel on one feature, i.e. $k(x,y)=\sum_{i=1}^{d}\beta_{i}x_{i}y_{i}$, the kernel selection task is exactly equivalent to our feature selection task and the results of (Gretton *et al.* 2012a) also hold for our approach. In the case of non-linear kernels, however, different aims are pursued. While we are finding a sparse set of features that best describe distributional differences, (Gretton *et al.* 2012b) optimize for a linear combination of kernels which maximizes test power. It, therefore, falls short of interpretability scenarios that are more complex than simple linear feature combinations.

We now proceed to illustrate how to perform feature selection with SpInOpt-MMD. The ultimate aim is to find a subset of the features of minimum cardinality which maximizes the change in the test statistic MMD. Notice that the objective function (4) gives higher (absolute) weight to features that differ between *X* and *Y* and lower (or zero) weight to features that do not vary. We could, therefore, select the features that have a non-zero weight as the ones that matter most. However, due to the finite sample size and the complex nature of the objective ℓ(X,Y;w), even unimportant features are never exactly zero. Therefore, we resort to a greedy, heuristic approach.

To obtain the distinguishing features, we first calculate the impact of a single feature on the MMD. Suppose the overall, optimized MMD is larger than a predefined significance threshold $c_{\alpha }$. If a feature is important, the MMD should decrease when dropping that feature, i.e. setting its weights to 0. If it is not, the MMD should not change, or even increase. We, therefore, in a univariate fashion, iterate through all features, dropping them consecutively, and calculate the impact on the MMD by computing the difference between the MMDs before and after dropping a feature. After this step, we start again by dropping the feature with the largest impact and append it to the list of selected features. We then re-evaluate if the MMD is still larger than $c_{\alpha }$. If so, we continue dropping features until ${\text{MMD}}_{\text{quad}}^{2}(\tilde{w}⊙X,\tilde{w}⊙Y)<c_{\alpha }$, where $\tilde{w}$ is the fitted weight $w^{*}$ with the weight of all selected features set to zero. By the end of the procedure, we obtain a set of selected features S and a set of not selected features I. Our approach is outlined in Algorithm 1. Our algorithm still employs a stepwise feature selection method but differs from the traditional forward or backward selection method (Kutner *et al.* 2005). Specifically, we select the most informative features that lead to the most MMD drop on the set of remaining candidate variables, rather than assessing performance changes on the set of selected variables in forward or backward selection. Furthermore, our selection criterion relies on a non-linear kernel MMD that measures the distributional discrepancy, rather than feature importance scores typically used in forward or backward selection methods. Song *et al.* (2012, 2007) briefly mentioned a similar selection criterion using MMD in Section 6.3 of Song *et al.* (2012), but did not further explore this idea.

Algorithm 1. Distinguishing features and feature pruning

**Input:** A two-sample dataset (X,Y), a weight vector w, a significance level α, (optional) a pruning percentage ρ

1: Sort w descending

2: Find top-ρ fraction of features, $f_{\rho }$, based on the top-ρ fraction of *w*

3: Calculate a significance threshold $c_{\alpha }$

4: Initialise S=∅

5: Compute the MMD on all features, ${\text{MMD}}_{\text{all}}$

6: **if**${\text{MMD}}_{\text{all}}<c_{\alpha }$**then**

7:   **return**S

8: **end if**

9: Set *D* to an empty list

10: **for**$f\in f_{\rho }$**do**

11:   Remove *f* from dataset

12:   Calculate the resulting ${\text{MMD}}_{\text{drop feature}}$

13:   Append ${\text{MMD}}_{\text{drop feature}}-{\text{MMD}}_{\text{all}}$ to *D*

14:   Reinsert *f*

15: **end for**

16: Sort *D* ascending

17: **for**d∈D**do**

18:   Remove the feature corresponding to *d*

19:   Add the feature to S

20:   Calculate the resulting ${\text{MMD}}_{\text{drop feature}}$

21:   **if**${\text{MMD}}_{\text{drop feature}}<c_{\alpha }$**then**

22:     **return**S

23:   **end if**

24: **end for**

25: **return**S

**Output:** A set S of selected features

As aforementioned, Algorithm 1 is a greedy approach. It is greedy because it selects one single most impactful feature at a time and it is heuristic because the stopping criterion $c_{\alpha }$ is fixed at the start of the selection procedure. In theory, one could obtain smaller feature sets by recalculating the significance threshold after each dropped feature, however, due to the expensive permutation approach in MMD-quad this would be computationally expensive. We highlight that although our approach appears to be univariate, it is fundamentally different from univariate feature selection and more aligned with contrast set learning (Bay and Pazzani 2001). In univariate feature selection, one tries to answer the question of whether a single feature is significant given no information about any other distributional features. Here, we ask, whether the distributional two-sample test is still statistically significant with respect to a predetermined threshold *given* a non-linear interaction of all remaining features. We argue that when the test fails to be statistically significant all distinguishing features (which could be interpreted as a contrast set) have been removed. As seen in Section Experimental Results, experiments confirm the validity of our heuristic.

The runtime complexity of Algorithm 1 with MMD-quad is O(dmn) and, thus, can be prohibitive for high-dimensional data. However, as a consequence of the sparsity-inducing regularization, the fitted vector $w^{*}$ will be approximately sparse (most values will approach zero). Therefore, most features will not have to be tested. To achieve performance improvements, we can threshold the weights and set all weights below a threshold to zero. This threshold naturally depends on the choice of regularization parameter λ. The number of features to be tested would also vary from sample to sample. To obtain predictable performance upgrades, we assume that, especially in high-dimensional settings, only a small fraction of the features is important to distinguish two distributions. Therefore, we can also set a parameter 0 < ρ ≤ 1 to only test a predefined fraction of the features. In this case, only the ρd largest features would be tested, resulting in a manageable runtime complexity of O(ρdmn). Note, that choosing a comparatively small ρ might result in missing distinguishing features, whereas a comparatively large ρ results in suboptimal runtime. Therefore, fine-tuning ρ (e.g. using a line search) can form a part of the hyperparameter tuning procedure. If further performance improvements are required, one can exchange the MMD-quad with the MMD-lin test, however, sacrificing test power.

Finally, we would like to point out that, similarly to other sparsity-based methods, our procedure may select different features in the case of highly correlated features. For example, in the case of two correlated discriminative features, the optimization algorithm may choose an optimum, where the first feature is selected and in a different run it may select the second feature. This stability issue is common to many feature selection algorithms Pes (2020) and, in our case, robustness may be achieved by repeating the optimization and Algorithm 1 multiple times with permuted *x* and *Y*, respectively.

## 3 Experimental setup

We demonstrate SpInOpt-MMD on a range of synthetic toy datasets and real-world biomedical applications in comparison to the classical MMD-quad, which is equivalent to setting all weights to one. We use an RBF kernel with the bandwidth parameter σ initialized to

(6) $$\sigma_{\text{med}}=\text{median}\left(\left\{||x_{i}\;-\;y_{j}||:i\in \{1,\ldots ,m\},\;\;j\in \{1,\ldots ,n\}\right\}\right).$$

For a fair comparison, we grid-search for the best kernel for the classical MMD-quad on the grid $[2^{-4},\ldots ,2^{4}]\sigma_{\text{med}}$. All datasets are subsampled to obtain an equal sample size for *X* and *Y* and the optimization is done on a training set of the input samples. We set aside 50% of the samples of *X* and *Y* for training and the other half for testing. To solve the Objective 4 we used L-BFGS due to its superior convergence rate over the gradient-based approach. The input gradients required for L-BFGS were computed with backpropagation using the PyTorch (Paszke *et al.* 2019) framework. All weights are initialized to the classical MMD, $w={\left(1,\ldots ,1\right)}^{T}$. Since, one optimal solution to Equation (4) would be to minimize $V[{\text{MMD}}_{\text{quad}}^{2}(w⊙X,w⊙Y)]$, we add a small regularization parameter $\lambda_{V}=10^{-8}$ to ensure numerical stability.

The null distribution ($H_{0}$) was calculated using a permutation approach where samples are randomly permuted between the groups and the resulting MMD is calculated. We use 400 permutations to approximate the null distribution. The significance threshold is set to α=0.01 in all experiments.

We also include the MMD-lin test as a second baseline. Both, the classic and the optimized version with feature weights are used. As mentioned above, SpInOpt-MMD transfers naturally from MMD-quad to MMD-lin. The null distribution of MMD-lin can be given in closed form (Gretton *et al.* 2012a), therefore, eliminating the need for computationally expensive permutations. However, this computational performance increase leads to a decline in test power.

As a third baseline, we calculate the statistical power using the two-sample test presented in (Jitkrittum *et al.* 2016). We use the original implementation (https://github.com/wittawatj/interpretable-test) with function tolerance of $10^{-4}$ and one test location. All other parameters were left at the default values.

### 3.1 Synthetic and benchmark data

Following Jitkrittum *et al.* (2016), we illustrate our approach on four toy synthetic data of Gaussian mixtures [the “same-gauss”, “mean-shift”, “variance-shift,” and “blobs” dataset of (Jitkrittum *et al.* 2016)], and two benchmark datasets comprising text data and face images [NeurIPS papers (Jitkrittum *et al.* 2016) and Karolinska faces(Lundqvist *et al.* 1998)]. Further details on these datasets can be found in the Supplementary Sections A–C.

### 3.2 Biomedical data

To illustrate the power of our approach in real-world data, we choose seven datasets in the biomedical domain across numerous modalities including medical imaging and omics data.

All included data were preprocessed by standard scaling.

#### 3.2.1 Imaging data and radiomic features

Imaging information is a pillar of modern noninvasive diagnosis and naturally comprises a broad variety of data types. Here we demonstrate SpInOpt-MMD to identify distinguishing features on 2D images and pre-extracted radiomic features. We include magnetic resonance images from the Alzheimer’s Disease Neuroimaging Initiative database (ADNI) of 203 Alzheimer’s diseased subjects and 163 healthy controls of comparable age (prevalence 0.55). All images were skull-stripped, intensity normalized, and co-registered to the MNI reference space (Horn 2016) [see (Brüningk *et al.* 2021) for image preprocessing details, data available upon request from adni.loni.usc.edu]. For each subject, we extracted 2D central coronal slices (193×193 pixels) as well as 1710 radiomic features [based on *pyradiomics* (van Griethuysen *et al.* 2017)] of the 3D left and right hippocampus—regions of the brain typically displaying Alzheimer’s disease associated brain tissue atrophy (Barnes *et al.* 2009). Additionally, we use radiomic features extracted from 3D T1 weighted MRIs of low-grade glioma patients acquired from The Cancer Imaging Archive [TCGA-LGG (Pedano *et al.* 2016, Kirby 2006, Clark *et al.* 2013) data available from https://www.cancerimagingarchive.net/collection/tcga-lgg, n=97] to stratify patients by the mutation status of the ATRX (prevalence 0.40) gene which is associated with treatment response implications (Nandakumar *et al.* 2017). The relevant gene mutation labels were inferred from corresponding whole genome sequencing data obtained from The Cancer Genome Atlas (TCGA, https://portal.gdc.cancer.gov/projects/TCGA-LGG).

#### 3.2.2 Gene expression data

In the area of multi-omics, efficient tools capable of handling high-dimensional data are key. We demonstrate use-case examples on two datasets including gene expression data (60k features) of the TCGA-LGG cohort (*n* = 97) aiming to predict ATRX mutation status and single-cell RNA sequencing data from PBMC from a Healthy Donor freely available from 10× Genomics (https://support.10xgenomics.com/single-cell-gene-expression/datasets/1.1.0/pbmc3k). For the latter, after preprocessing (Satija *et al.* 2015) we retained 2638 cells with 1838 features each allocated to one of eight cell types using Leiden clustering (Traag *et al.* 2019) (see Supplementary Fig. S9).

#### 3.2.3 Other omics data

A last set of experiments was conducted on matrix-assisted laser desorption ionization time of flight (MALDI-TOF) spectra (binned to 6000 features) for the prediction of antimicrobial resistance in two bacterial species obtained from the Database of Resistance Information on Antimicrobials and MALDI-TOF Mass Spectra (DRIAMS) (Weis *et al.* 2022b, data available at Weis *et al.* 2022a). Specifically, this included *Staphylococcus aureus* resistance to Oxacillin (*n* = 1204, prevalence 0.85, DRIAMS-SA), and Ceftriaxone resistance in *E. coli* (*n* = 1386, prevalence 0.82, DRIAMS-EC).

#### 3.2.4 Evaluation procedure

For each dataset, we use both SpInOpt-l(inear) and SpInOpt-q(uadratic) (For notational convenience we drop the “-MMD” from the notation as there is no ambiguity.) to identify distinguishing features. Given the limited sample sizes encountered in these real-world examples, we did not perform further hyperparameter tuning of λ on a separate data split but instead opted for a fixed λ=0.1d to ensure sufficiently large test datasets. Results for test power, number of selected features, and obtained type I errors are given as a function of λ in the Supplementary Section E. We repeat each experiment on radiomic and MALDI-ToF data 500 times. Due to computational constraints, each SpInOpt-q on expression and imaging data is run 100 times. In each experiment, we randomly subsample the data to ensure equal sample sizes for *X* and *Y* and randomly partition them into train and test sets.

In the absence of ground truth, we rely on qualitative proxies to evaluate the efficacy of the feature selection and assess the identification of established imaging biomarkers. For a semi-quantitative comparison, we benchmark our approach against standard feature selection and interpretability algorithms in a classification setting.

For tabular omics data with binary labels (ADNI radiomic, TCGA-LGG expression and radiomic, DRIAMS-SA, DRIAMS-EC), we evaluate the performance of a logistic regression classifier on all or preselected features. We compare SpInOpt features to features ranked using

- SHapley Additive exPlanations **(SHAP)**: using the *shap* librarywe calculate SHAP-values for all contributing features.
- univariate feature selection (**univar. FS.**): we perform a univariate association analysis to obtain *P*-values for each feature.

From these rankings, three sets of highest-ranking features are selected. Two sets using the same number of features as identified by SpInOpt [“univar. FS. (fixed #),” “SHAP (fixed #)”], and a third set using all statistically significant features (accounting for multiple testing corrections by Bonferroni) identified by univariate association analysis [univar. FS. (significant)]. The selected (or all) features are provided to the logistic regression classifier. We further perform a grid search on the training data over both L1 and L2 regularization strengths (ranging from $10^{-7}$ to $10^{4}$) implying that the baseline using all features as input is subjected to optional feature selection. We score classification performance by auROC.

In addition to classifier performance we report the fractions of SpInOpt-q features overlapping with SHAP (fixed #), univar. FS. (fixed #), and univar. FS. (significant), as well as the fraction of univar. FS. (significant) features that are part of the SpInOpt-q features.

For PBMC data, we compare cluster marker gene analysis based on *t*-tests (using the *rank_genes_groups* function of the python *scanpy* package) with the SpInOpt-identified important features as the overlap with the top ten marker genes and the top ten MMD selected genes. This evaluation aims to provide a notion of the usefulness of the identified distinguishing features only. In practice, in-depth evaluation of the identified features through, e.g. comparison with canonical markers can reveal the biomedical importance of these features.

For 2D imaging data (ADNI-2D), a shallow 2D convolutional neural network (CNN, 2 layers, 64 hidden dimensions, and 7 kernel sizes) was used to classify based on either full images or on images with all MMD-unimportant features being masked. We evaluate classification performance (over 5-fold cross-validation) in addition to the qualitative distribution of the identified significant image pixel locations.

## 4 Experimental results

### 4.1 Synthetic and benchmark data

Given the choice of biologically relevant sample sizes (order of hundreds) and dimensions (order of thousands), the results on the synthetic data differ largely from Jitkrittum *et al.* (2016) for larger sample sizes. None of the methods presents sufficient statistical evidence to reject the null hypothesis in the same-gauss dataset yet the chosen sample sizes were too small for any test to reliably distinguish the more subtle distribution differences encountered in the variance-shift and blobs datasets. All numerical results can be found in the Supplementary data.

The mean-shift dataset showed good performance and in Supplementary Figs S1–S7, we summarize the statistical power of both optimized and non-optimized MMD tests with respect to varying dimensions, sample size, and lambda. While SpInOpt-q often obtains maximal test power, the test power of SpInOpt-l is also improved over its equal feature weight baseline. However, the performance of SpInOpt-l declines more rapidly as the number of data points decreases. The feature-selection properties of SpInOpt-q are near-optimal with type I errors only present in the regime λ > 0.1n and few type II errors (see Supplementary data for further details).

On the Karolinska faces dataset (Table 1), the baseline MMD-q test already achieves maximum test power, therefore, any optimization would only reflect in a potentially lower *P*-value. However, the power of the MMD-l test almost doubles with our optimization procedure over the unomptimized baseline MMD tests. The most discriminative face image pixels for positive and negative emotions can be found in Supplementary Fig. S8. In agreement with previous work (Jitkrittum *et al.* 2016), these are located within the eyes and the top of the nose. In contrast to the test presented in (Jitkrittum *et al.* 2016), our method also allows us to quantify the importance of the features and the feature selection is sparse.

**Table 1.** The type I errors (“± versus ±”) and statistical power of the baseline and SpInOpt-q and SpInOpt-l methods rounded to three figures.

Problem	MMD-q	MMD-l	SpInOpt-q	SpInOpt-l	**Jitkrittum *et al.* (** 2016 **)**
± versus ±	0.004	0.006	0.012	0.010	0.010
+ versus −	**1.000**	0.480	**1.000**	0.828	0.998

For comparison, we also restate the results of Jitkrittum *et al.* (2016). The suffix “-q” denotes MMD-quad and “-l” MMD-lin. The bold values indicate the highest test powers obtained.

In the NeurIPS papers dataset, SpInOpt-q also increases statistical power in every scenario and achieves maximal test power in six out of seven paper categories tested. The statistical power or, respectively, type I errors on the NeurIPS dataset are given in Supplementary Table S2. We further list the top 10 most discriminative words of the NeurIPS datasets in the Supplementary data. These are the selected features in this dataset.

### 4.2 Biomedical data

#### 4.2.1 Test performance

**Figure 1.**
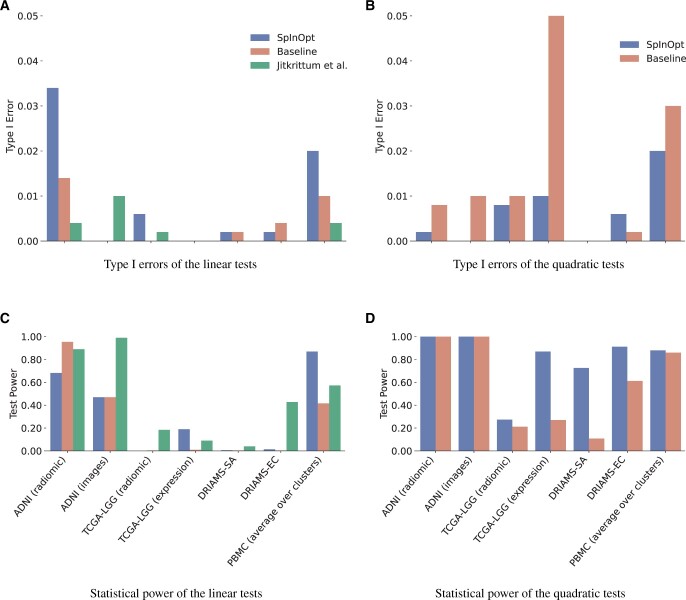
The type I errors and statistical power of SpInOpt and their unoptimized counterparts (“Baseline”). We also report the results of Jitkrittum *et al*. (2016), however, since (Jitkrittum *et al*. 2016) is a *linear* method, the column is omitted in the quadratic tests. For conciseness, we average the test power over all eight clusters on the PBMC dataset. The suffixes are “-SA”: *Staphylococcus aureus* and “-EC”: *Escherichia coli*.

The statistical power and type I errors of the two unoptimized baseline MMD methods, the Jitkrittum *et al.* (2016) test, and the corresponding SpInOpt-MMD tests for λ = 0.1 can be found in Fig. 1 (see Supplementary data for a tabular representation and the full hyperparameter sweep results). All tests were able to correctly reject the null hypotheses $H_{0}$ with a type I error around the pre-defined false discovery rate of α=0.01. In the testing scenarios, the linear MMD tests performed substantially worse than their quadratic counterparts. This is not unexpected as the sample sizes are small (order of tens to one hundred) compared to the number of features (order of thousands).

The SpInOpt-q is the best-performing method in six out of the seven datasets studied. In particular, it achieves large increases in test power in high-dimensional tabular datasets, such as gene expression or MALDI-ToF data. We attribute this in part to the sparsity of the optimized features, which allows the test to focus on the most distinguishing distribution features only. No test was performed satisfactorily on the small radiomic TGCA-LGG dataset, whereas both modalities of the ADNI dataset are distinguishable by MMD-q and SpInOpt-q, with MMD-l and SpInOpt-l also showing optimal test power on the radiomic features. We also report the results of the kernel-based test of Jitkrittum *et al.* (2016) whenever obtainable. Due to this method being a linear method, we report it in the linear column, although this constitutes a slight abuse of notation. Note that for our datasets the high-dimensional ADNI image and LGG expression datasets experienced memory issues. The ADNI radiomic and DRIAMS datasets suffered from convergence issues and, therefore, we can only report results on the PBMC and radiomic LGG datasets. In both cases, SpInOpt-q outperforms the method of Jitkrittum *et al.* (2016).

The variable performance of all tests on the PBMC dataset is driven by cluster size. For five clusters SpInOptMMD-q exhibited a test power of one, whereas two clusters (six and seven) only achieved 0.20 and 0.26, respectively. We attribute this to our experimental design of subsampling the larger group in the dataset to the number of samples in the smaller. Clusters six and seven, are small ($n_{\mathrm{cluster}6}=35$, $n_{\mathrm{cluster}7}=13$) in comparison to the rest of the dataset ($n_{\mathrm{rest}}=432(164,1124)$, mean with full range), which results in small train and test sets, thus, sacrificing test power.

#### 4.2.2 Evaluation of selected features

For our five binary class tabular datasets [i.e. ADNI (radiomic), TCGA-LGG (radiomic), TCGA-LGG (expression), DRIAMS-SA, DRIAMS-EC] prediction performance as a proxy for the relevance of the identified biomedical features selected by either univar. FS, SHAP, or SpInOpt-q is given in Table 2 (see Supplementary Table S3 for corresponding results with SpInOpt-l). We compare based on either equal numbers of features, emphasizing the rank of the identified features, or based on all selected features for univariate association analysis. In line with our result on test power, we observe competitive performance for SpInOpt-based feature selection across four out of five datasets for SpInOpt-q. SpInOpt-l was outperformed by other feature selection methods [ADNI (radiomic), DRIAMS-SA, DRIAMS-EC], or did not yield any selected features [TCGA-LGG (radiomic)]. Importantly, for the TCGA-LGG expression and radiomic feature sets, SpInOpt-q could identify important features that improved prediction performance over the baseline, whereas univariate association did not identify any.

**Table 2.** Logistic regression binary classification performance (ROCAUC) based on different experimental datasets and feature selection approaches as indicated based on the same number of features as identified by SpInOpt-q.

Dataset	All features	univar.FS	univar.FS	SHAP	SpInOpt-q
		(fixed #)	(significant)	(fixed #)	
ADNI	0.91±0.05	**0.93** ± **0.04**	0.02±0.04	0.91±0.04	0.92±0.03
(radiomic)					
TCGA-LGG	0.77±0.13	0.78±0.15	–	0.78±0.14	**0.79** ± **0.12**
(radiomic)					
TCGA-LGG	0.89±0.09	0.90±0.03	–	0.88±0.07	**0.96** ± **0.03**
(expression)					
DRIAMS-SA	0.90±0.03	0.89±0.04	0.75±0.04	0.89±0.04	**0.92** ± **0.03**
DRIAMS-EC	0.85±0.02	0.86±0.02	0.85±0.03	0.84±0.02	**0.88** ± **0.02**

Results are reported as mean values and standard deviation over all cross-validation folds. Univar.FS, feature selection by univariate association; (significant), using all significant features; (fixed #), using a fixed number of features equal to the number of selected SpInOpt-q features; –, no selected features found. The bold values indicate the highest test powers obtained.

**Figure 2.**
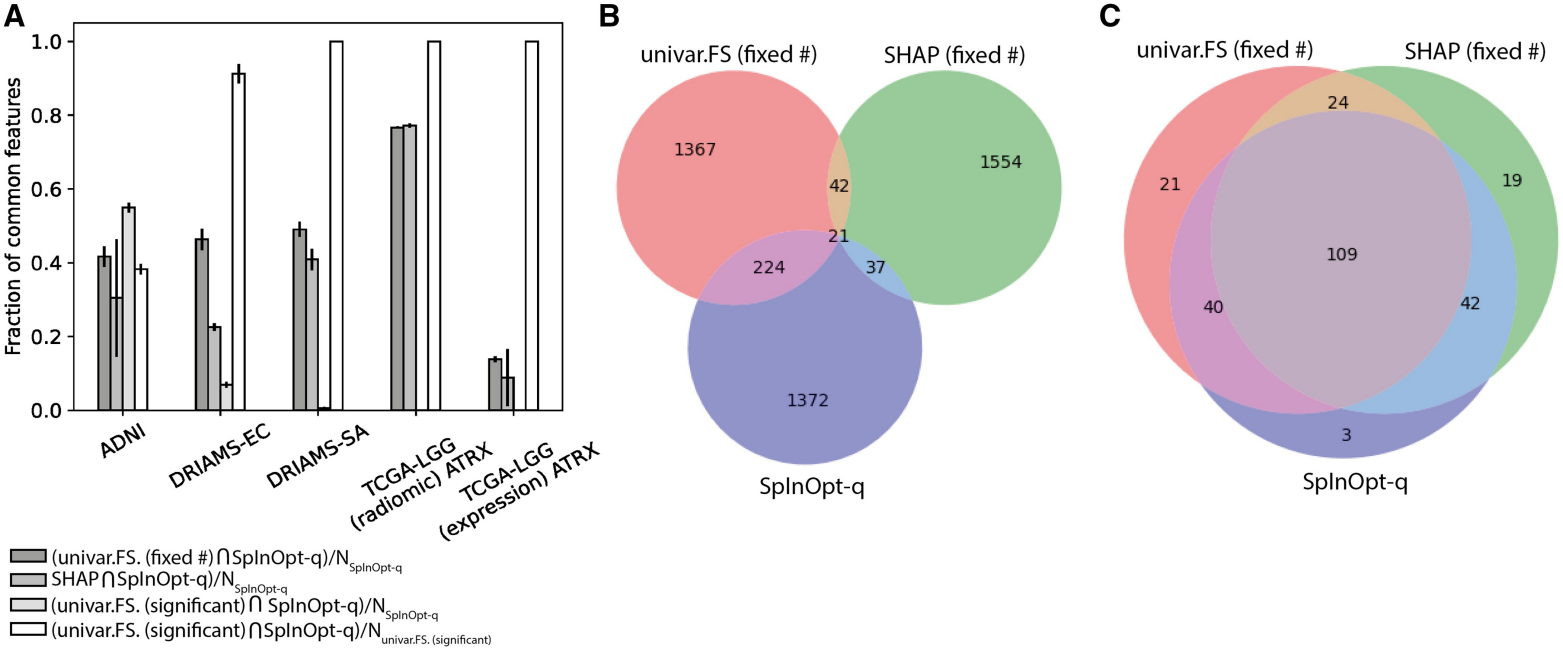
(A) Summary of the relevant fractions of overlapping features for the included binary classification datasets. Barplots indicate the fraction as mean values and standard deviation across cross-validation folds. Fractions are reported relative to the number (*N*) of features of either SpInOpt-q or all statistically significant features identified by univariate feature selection. (B, C) Representative examples of Venn diagrams showing the overlap between the identified features between SpInOpt-q, univariate feature selection [univar.FS (fixed#)], and SHAP analysis for one cross-validation fold in the TCGA-LGG expression (B) and radiomic (C) feature set.

When looking at the overlapping fraction in the relevant features, shown in Fig. 2A we observe that each of the three feature selection methods yields in part different features. Generally, there is a stronger overlap between univar. FS (fixed #) and SpInOpt-q selected features than between SHAP and SpInOpt-q features. For most datasets, the statistically significant (as by Bonferroni corrected 0.05 significance threshold) features identified by univariate association analysis were a subset of the SpInOpt-q features. However, the overall feature overlap for all methods varies strongly depending on the relevant datasets.

For the multiclass PBMC dataset, we observe an agreement for a fraction of 0.64±0.23 and 0.55±0.30 between the top ten marker genes and the MMD selected features across cell types for SpInOpt-q and SpInOpt-l, respectively, indicating the identification of relevant features. We observe the strongest variation in the largest cluster 0.

### 4.3 Image data

**Figure 3.**
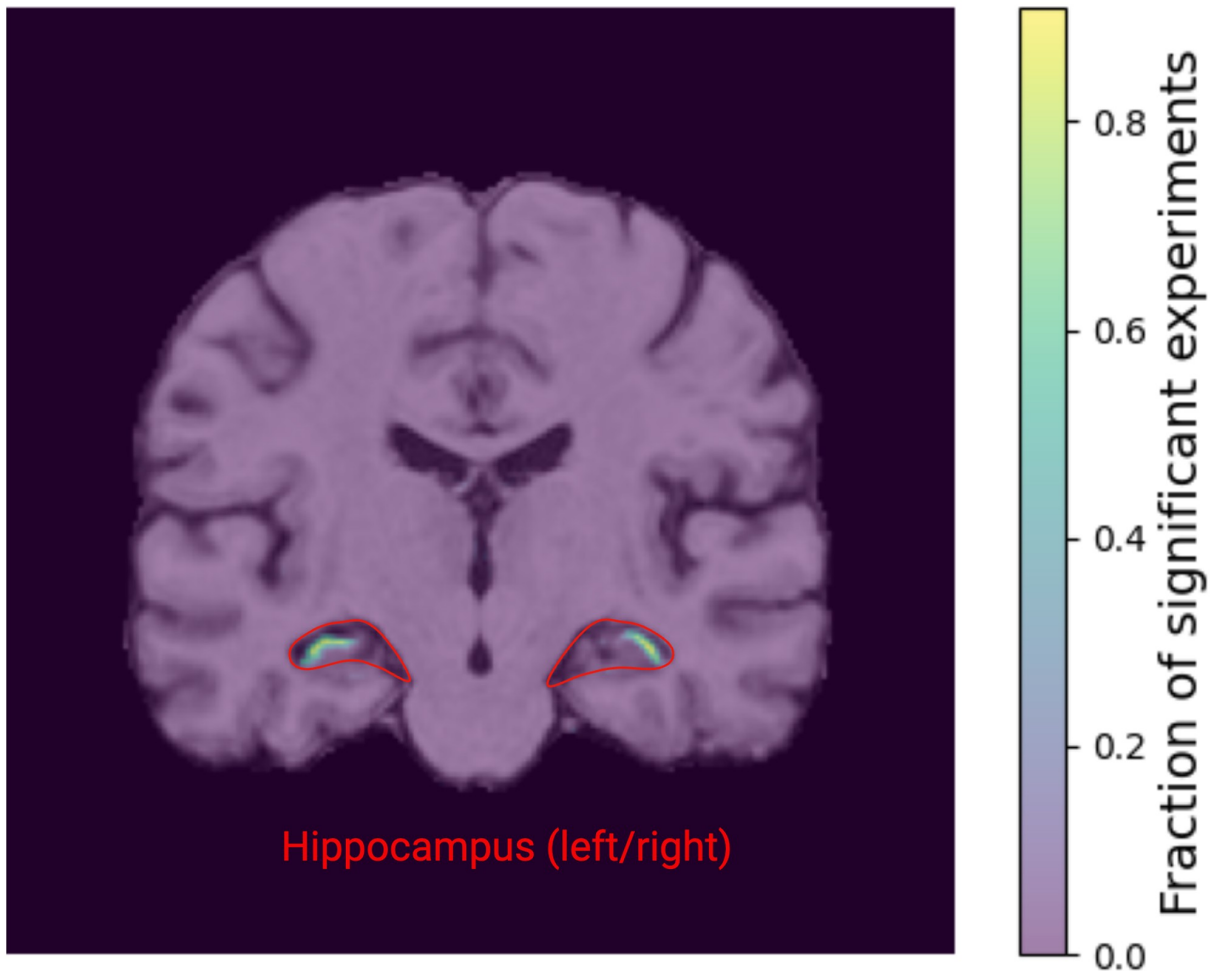
Overlay of the identified significantly distinguishing image pixels (heatmap) with one of the used MR image slices of a healthy subject. The left and right hippocampus are contoured in red. SpInOpt-q identifies Alzheimer’s disease-associated regions to be located within the left and right hippocampus as expected.

In magnetic resonance images of Alzheimer’s diseased and healthy patients our algorithm picks up image features predominantly located in the left and right hippocampus (see Fig. 3). These regions align well with the known imaging biomarkers of Alzheimer’s disease (Brüningk *et al.* 2021). This result is further supported by the improved classification performance when using only the SpInOpt-q-identified image pixels as input to a convolutional neural network, yielding an area under ROC (auROC) of 0.85±0.04, with an improvement of 4% compared to the same model using the entire image as input (0.81±0.05). This result is auspicious as SpInOpt-q may be seen as a computationally efficient alternative to CNN-interpretability analysis-based identification of imaging biomarkers. This allows for direct statistical evaluation rather than arbitrary scales used in saliency maps.

## 5 Conclusion

In this article, we close the gap between global two-sample testing and local feature selection by introducing SpInOpt-MMD. SpInOpt-MMD goes beyond the classical MMD-based two-sample tests, gaining interpretability by assigning and optimizing sparse feature weights. These feature weights can be used to obtain binary feature masks of important and unimportant features.

In our experiments, we show that SpInOpt-MMD always exhibits greater test power than the unoptimized MMD tests. We furthermore show that superior statistical power are achieved in comparison to the interpretable two-sample test introduced in Jitkrittum *et al.* (2016).

We further investigated the ability of SpInOpt-MMD to select informative features by studying downstream classification tasks on the image, radiomic, expression, and MALDI-ToF datasets considered. We showed that a classifier using SpInOpt-q selected features achieves superior classification performance in all but one dataset (ADNI radiomic) considered. This indicates that not only is SpInOpt-MMD a superior two-sample test, but it is also an efficient way of selecting informative features.

There are theoretical open questions not yet discussed in this work, for example under which conditions the optimization problem (4) is convex in each orthant. A generalization of our approach to deep kernels as introduced by Liu *et al.* (2020) would also be highly relevant. On the application side, due to the kernel formulation, SpInOpt-MMD can be applied to any data modality in which the similarity can be described by a differentiable characteristic kernel. Therefore, future research would include expanding our approach to structured data such as graphs and time series. Furthermore, owing to the stepwise and greedy nature of SpInOpt-MMD, recent techniques for multiple testing and selective inference (Taylor and Tibshirani 2015) could be used to enhance the method. These methodologies effectively tackle the challenge of correcting for the effects of multiple comparisons and selection biases. Detailed studies of particular datasets, for example, single-cell datasets, and comparisons with the state-of-the-art methods in the specific field (e.g. marker gene selection in single-cell data) is a possible future direction. To summarize, we have here demonstrated the utility of SpInOpt-MMD on a large variety of data modalities and also pointed out several kernels that can be used. We are convinced that this work would be a valuable contribution not only to the two-sample testing but also to the feature selection literature.

## Supplementary Material

btae251_Supplementary_Data

## Data Availability

We added specific data availablity links for all of the used data sets which are publicly available upon request from these sources.
