# Supplementary material for: Biomarker identification by interpretable maximum mean discrepancy
Source: Bioinformatics. 2024 Jun 28;40(Suppl 1):i501–10. doi: 10.1093/bioinformatics/btae251 (PMC11211810; doi:10.1093/bioinformatics/btae251)
Supplement: btae251_Supplementary_Data [file btae251_supplementary_data.pdf]

Supplementary Information for Biomarker identification by interpretable Maximum Mean Discrepancy

Michael F. Adamer[1,4], Sarah C. Brüningk[1,2,4], Dexiong Chen[1,3,4], and Karsten Borgwardt[1,3,4]

[1]*Department of Biosystems Science and Engineering, ETH Zurich, Mattenstr. 26, 4058 Basel, Switzerland*
[2]*Department of Health Sciences and Technology, ETH Zurich, Lengghalde 2, 8008 Zurich, Switzerland*
[3]*Department of Machine Learning and Systems Biology, Max Planck Institute of Biochemistry, Am Klopferspitz 18, 82151 Martinsried, Germany*
[4]*Swiss Institute for Bioinformatics (SIB), Amphipôle, Quartier UNIL-Sorge, 1015 Lausanne, Switzerland*

A Synthetic Gaussian Mixtures

In this section we provide the details on our synthetic experiments.

A.1 Experimental Setup

All experiments are repeated $n = 500$ times to estimate the test power. At the start of each experiment, a new sample of each distribution is generated and they are partitioned randomly into train and test sets. In the first three experiments, we vary the data dimension, the sample size, and the regularisation parameter respectively in ranges that are typically encountered in biomedical scenarios. In particular, we vary the dimension from 5 to 1500 and the sample size to 200 to 1000. The regularisation parameter λ is varied from 10^{-3} to 10^3. We report the test power or type I error and, since a ground truth of distinguishing features is known, we also calculate the type I and type II errors on the selected features. All features are selected based on a $\rho = 0.1$ pruning.

The first dataset ("same-gauss") consists of two isotropic Gaussians with mean zero and identity covariance matrix. In this case, the null hypothesis holds and the type I error should stay at the pre-specified level of α.

The second dataset ("mean-shift") consists of one Gaussian with mean $\mu_1 = (1, 0, 0, 0, \dots)^T$ and identity covariance matrix and one Gaussian with mean zero and also identity covariance matrix. In this scenario, our MMD test should identify the feature along the first dimension as significant.

The third dataset ("variance-shift") consists of two mean zero Gaussians with covariance matrices $\Sigma_1 = \text{diag}(2, 1, 1, 1, \dots)$ and $\Sigma_2 = \text{diag}(1, 1, 1, 1, \dots)$ respectively. Again, the most distinguishing feature is along the first dimension.

Finally, we also include the "blobs" dataset of [1, 2]. This consists of two two-dimensional Gaussian mixtures arranged on a 4×4 grid. The distinguishing transformation between those distributions is that the covariance matrix of the first distribution (P) has eigenvalues of 2.0, whereas the covariance matrix of Q has eigenvalues of 1.0. The blobs dataset encodes much more subtle, local differences rather than global differences and it has been shown already in [3] that the classical MMD-quad and MMD-lin exhibit comparatively low test power.

A.2 Results

Figure 1 treats the same-gauss dataset and Figures 2, 4, 6 highlight our results on the variance-shift, mean-shift, and blobs datasets respectively. The reported runtimes are composed of the optimisation time, where applicable, and the test time. Since we report both, the runtimes for SpInOpt-MMD and the non-optimised baseline MMD, the optimisation overhead can be estimated from the plots. All runtime calculation were done on an Intel Core i7-10700K CPU with 3.80 GHz, 16 processor cores and 32 GB RAM. Our results on feature selection can be found in Figures 3, 5, and 7.

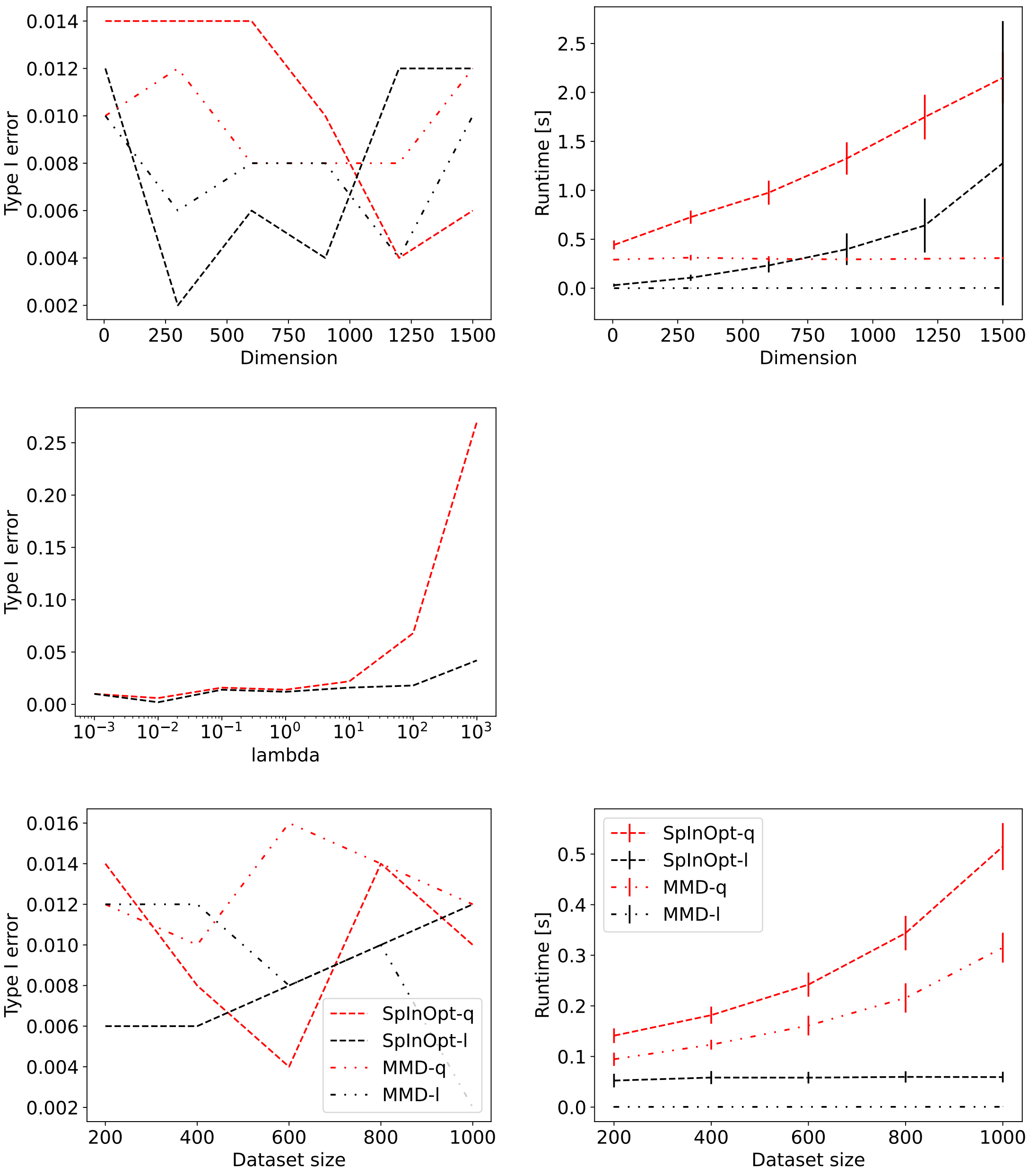

(a) Type I error (b) Runtime

Figure S 1: Results on the H_0 same-gauss dataset.

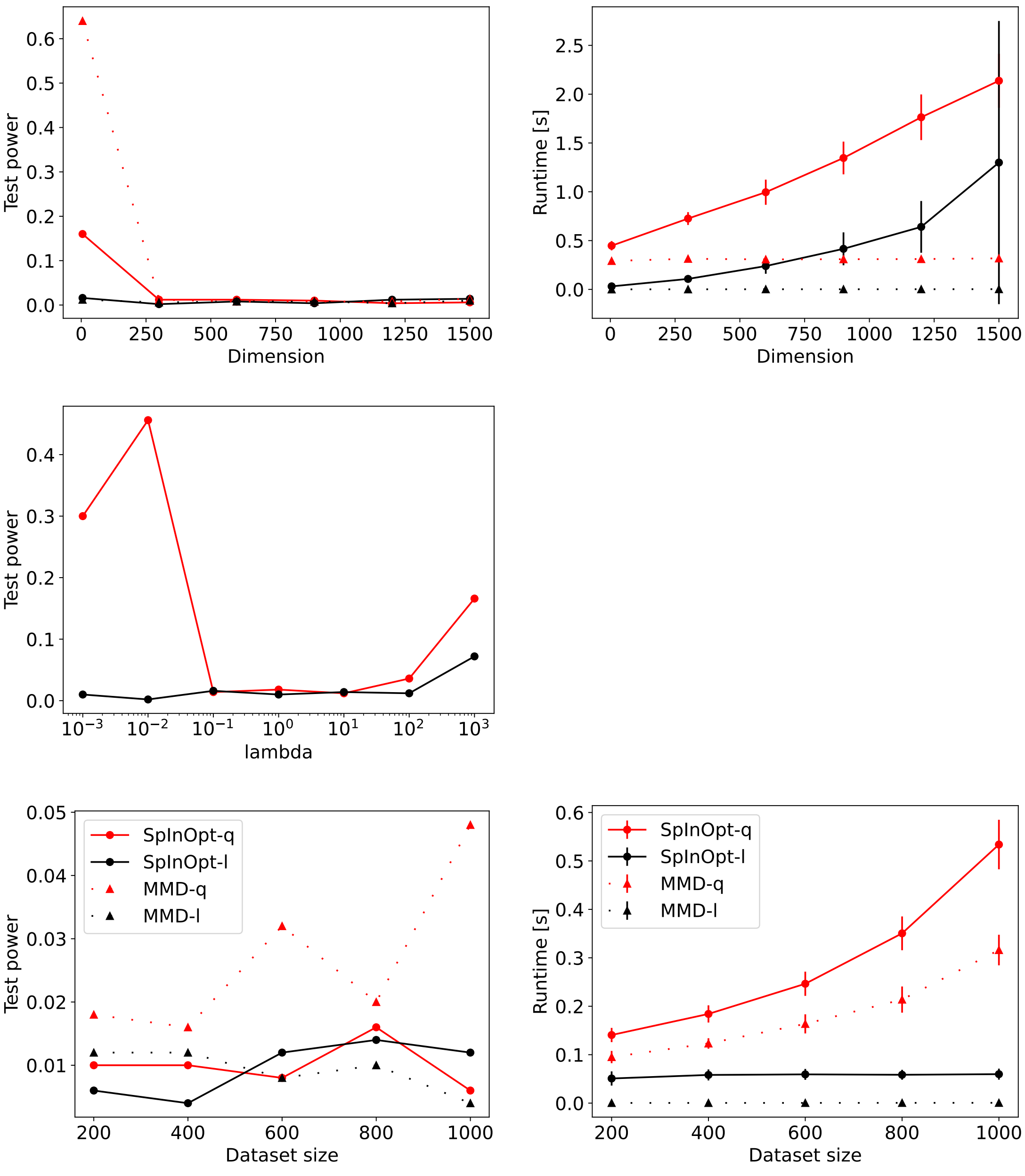

(a) Test power (b) Runtime

Figure S 2: Results on the variance-shift dataset.

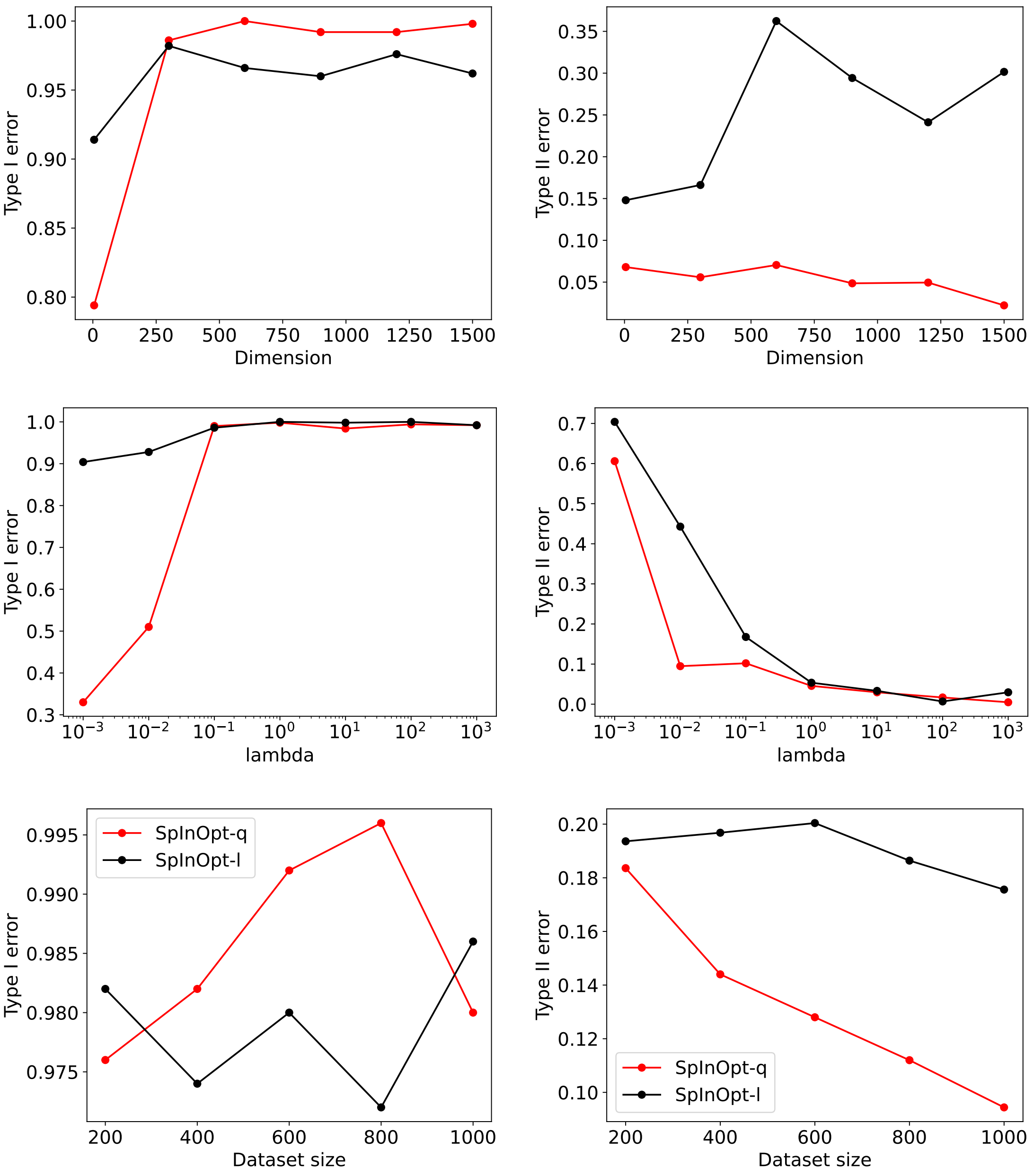

(a) Type I error

(b) Type II error

Figure S 3: Type I and II errors of the feature selection on the variance-shift dataset.

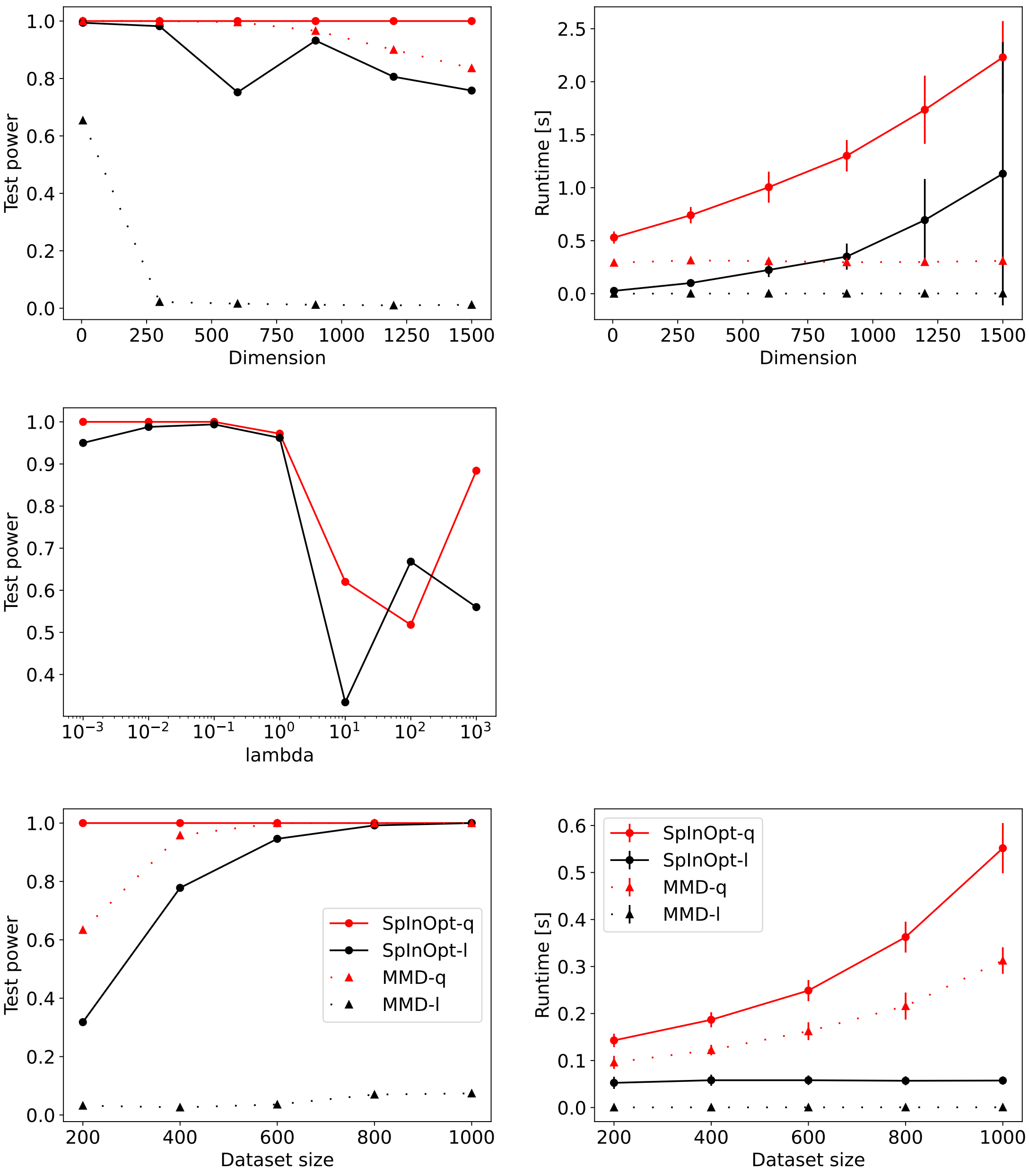

(a) Test power (b) Runtime

Figure S 4: Results on the mean-shift dataset.

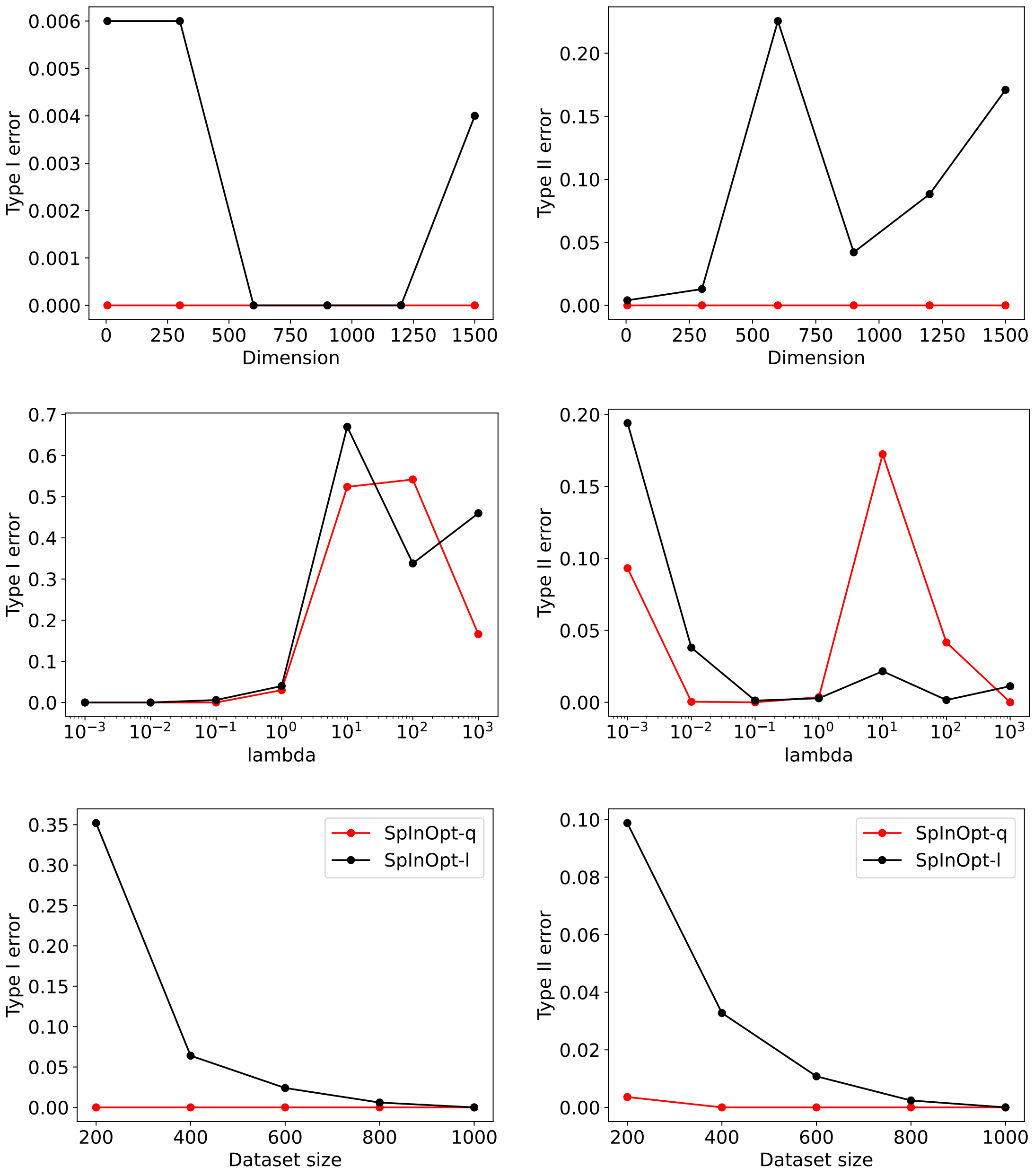

(a) Type I error

(b) Type II error

Figure S 5: Type I and II errors of the feature selection on the mean-shift dataset.

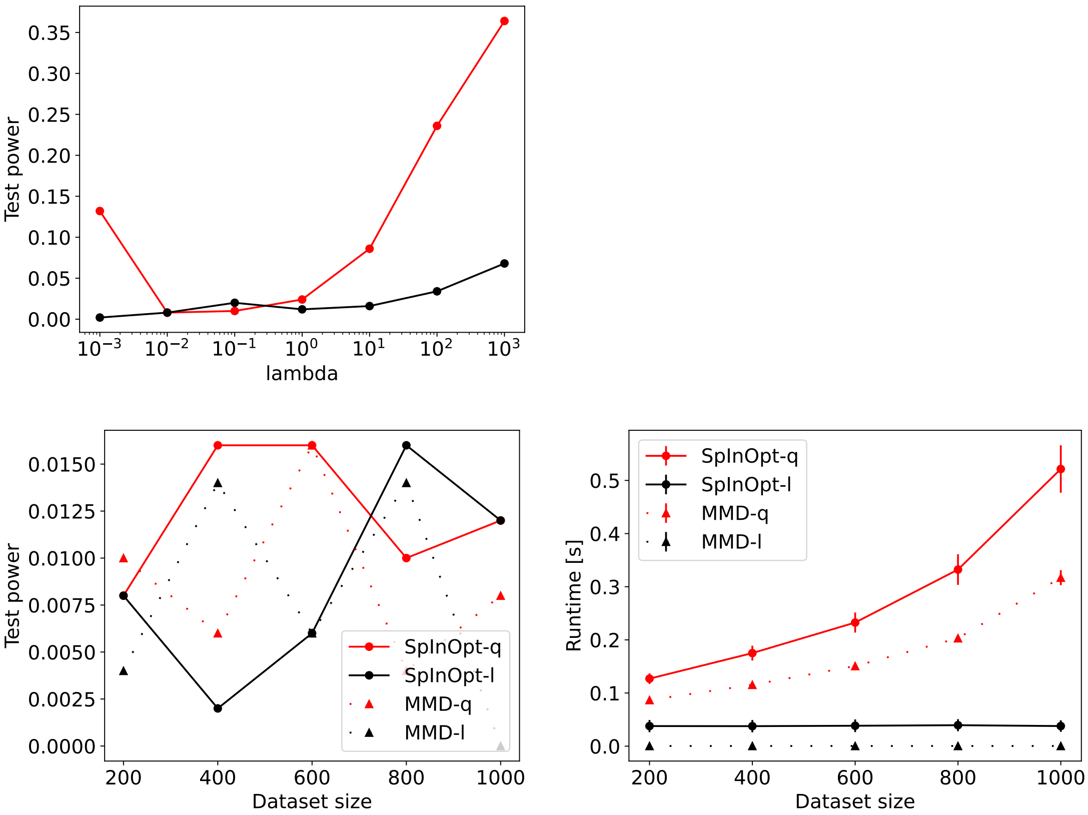

(a) Test power

(b) Runtime

Figure S 6: Results on the blobs dataset.

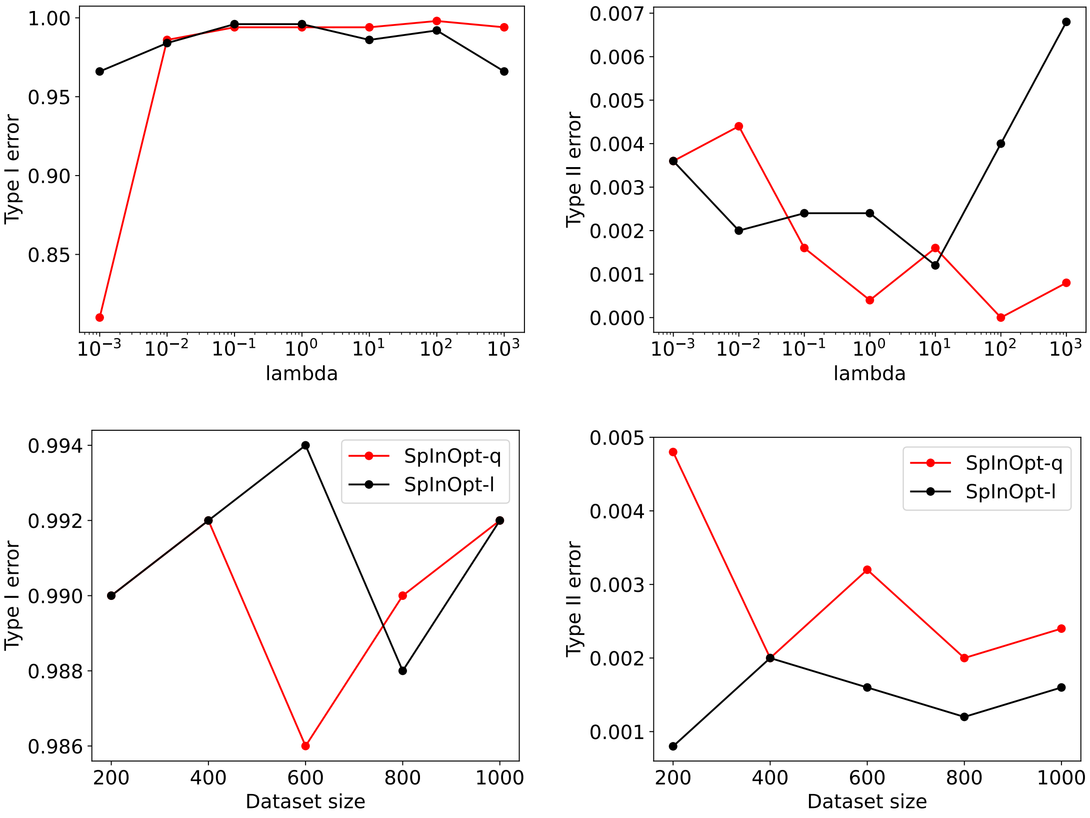

(a) Type I error

(b) Type II error

Figure S 7: Type I and II errors of the feature selection on the blobs dataset.

B Karolinska faces dataset

We tested our approach on the Karolinska faces dataset [4] with regularisation parameter $\lambda = 0.1 n_{\text{pixels}}$. In alignment with the experimental protocol of Jitkrittum et al. [3], each experiment was repeated 500 times. The test powers and type I errors respectively can be found in Table 1. The baseline MMD-q test already achieves maximum test power, therefore, any optimisation would only reflect in a potentially lower p-value. The power of the MMD-l test almost doubles to 0.828.

**Problem**	**MMD-q**	**MMD-l**	**SpInOpt-q**	**SpInOpt-l**	**Jitkrittum et al.** [3]
$\pm$ vs. $\pm$	0.004	0.006	0.012	0.01	0.01
$+$ vs. $-$	**1.0**	0.48	**1.0**	0.828	0.998

Table 1: The type I errors ("$\pm$ vs. $\pm$") and test powers of the baseline and SpinOpt-q and MMD-l methods rounded to three significant figures. For comparison, we also restate the results of Jitkrittum et al. [3]. The suffix "-q" denotes MMD-quad and "-l" MMD-lin.

The most discriminative features can be found in Figure 8. As already mentioned in [3], the most discriminative features are the eyes and the top of the nose. In contrast to the test presented in Jitkrittum et al. [3], our method also allows us to quantify the importance of the features and the feature selection is sparse.

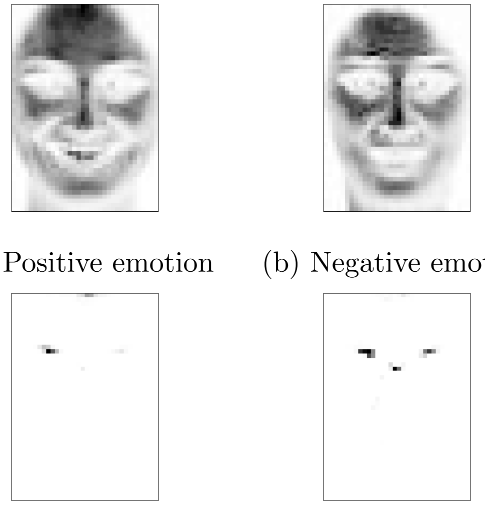

(a) Positive emotion (b) Negative emotion

(c) SpInOpt-l (d) SpInOpt-q

Figure S 8: Examples of the Karolinska face dataset [4].In the second row, we plotted the most discriminative pixels as found by SpInOpt-MMD with linear and quadratic tests respectively. The pixel colour is an indicator of how often said pixel was identified as important.

C NeurIPS papers dataset

We also included the NIPS dataset first studied in [3]. Again, we kept the regularisation parameter as $\lambda = 0.1 n_{\text{words}}$. In alignment with the experimental protocol of Jitkrittum et al. [3], each experiment was repeated 500 times. The test powers and type I errors respectively can be found in Table 2. The optimised MMD-quad provides a significant increase in test powers in every scenario and achieves maximal test power in six out of seven datasets tested (remember "bayes-bayes" represents H_0).

**Problem**	**MMD-q**	**MMD-l**	**SpInOpt-q**	**SpInOpt-l**	**Jitkrittum et al.** [3]
bayes-bayes	0.01	0.01	0.01	0.00	0.012
bayes-deep	0.75	0.43	0.87	0.28	**0.95**
bayes-learning	**1.00**	0.18	**1.00**	0.43	0.99
bayes-neuro	**1.00**	0.98	**1.00**	0.99	**1.00**
deep-learning	0.64	0.67	**1.00**	0.08	0.96
deep-neuro	0.80	0.44	**1.00**	0.42	0.40
neuro-learning	**1.00**	0.53	**1.00**	0.73	0.96

Table 2: The type I errors ("bayes-bayes") and test powers of the baseline and SpinOpt MMD-quad and MMD-lin methods rounded to three significant figures. For comparison, we also restate the results of [3]. The suffix "-q" denotes MMD-quad and "-l" MMD-lin.

The top discriminative words in each dataset are as follows.

- SpInOpt-l:
  - **bayes-bayes**: essay, establish, esther, eta, evas, essenc, densiti, reward, imag, posterior
  - **bayes-deep**: exponenti, classifi, boltzmann, templat, game, action, net, imag, neuron, infer
  - **bayes-learning**: boost, segment, game, bandit, human, graph, graphic, classifi, markov, infer
  - **bayes-neuro**: eras, era, equiti, equilibr, equilater, epilept, ethnic, typefac, circuit, spike
  - **deep-learning**: boltzmann, entropi, inequ, gibb, convex, deep, imag, loss, recurr, lemma
  - **deep-neuro**: expans, expend, expert, expertis, explan, exploit, expedit, boltzmann, spike, convolut
  - **neuro-learning**: expertis, expend, expert, recognit, cortex, polici, hypothesi, transistor, stimulus, theorem
- SpInOpt-q:

- **bayes-bayes**: graphic, convex, track, chain, rank, asymptot, lift, label, polici, bay
- **bayes-deep**: motif, adaptor, imag, covari, boltzmann, mont, graphic, neuron, net, infer
- **bayes-learning**: extra, typefac, inequ, carlo, polynomi, hypothesi, classifi, graphic, markov, infer
- **bayes-neuro**: eras, era, equiti, equilibr, equilater, epilept, epidemiolog, eta, typefac, spike
- **deep-learning**: digit, delay, recurr, deep, loss, inequ, imag, recognit, convex, lemma
- **deep-neuro**: fire, infer, pool, discrimin, recurr, face, cell, convolut, boltzmann, spike
- **neuro-learning**: exploit, exposur, extinguish, extract, expos, typefac, stimulus, hypothesi, proof, theorem

D Biomedical Data sets - additional results

**Dataset**	**Method**	$\boldsymbol{H_0}$**-q**	$\boldsymbol{H_0}$**-l**	**Test-q**	**Test-l**
ADNI	Baseline	0.008	0.014	**1.000**	0.954
(radiomic)	Jitkrittum et al. [3]	-	0.004	-	0.890
	SpInOpt	0.002	0.034	**1.000**	0.682
ADNI	Baseline	0.010	0.000	**1.000**	0.470
(images)	Jitkrittum et al. [3]	-	0.010	-	0.990
	SpInOpt	0.000	0.000	**1.000**	0.470
TCGA-LGG	Baseline	0.010	0.000	0.212	0.004
(radiomic)	Jitkrittum et al. [3]	-	0.002	-	0.184
	SpInOpt	0.008	0.006	**0.274**	0.000
TCGA-LGG	Baseline	0.050	0.000	0.270	0.010
(expression)	Jitkrittum et al. [3]	-	0.000	-	0.090
	SpInOpt	0.010	0.000	**0.870**	0.190
DRIAMS - SA	Baseline	0.000	0.002	0.108	0.004
	Jitkrittum et al. [3]	-	0.000	-	0.040
	SpInOpt	0.000	0.002	**0.726**	0.006
DRIAMS - EC	Baseline	0.002	0.004	0.612	0.004
	Jitkrittum et al. [3]	-	0.000	-	0.428
	SpInOpt	0.006	0.002	**0.912**	0.014
PBMC	Baseline	0.030	0.010	0.860	0.416
(average over	Jitkrittum et al. [3]	-	0.004	-	0.573
clusters)	SpInOpt	0.020	0.020	**0.880**	0.870

Table 3: The type I errors (H_0) and test powers of the unoptimised baseline and SpInOpt-MMD methods rounded to three figures. We also report the results of [3], however, since Jitkrittum et al. [3] is a *linear* method, the column is omitted in the quadratic tests. For conciseness, we average the test power over all 8 clusters on the PBMC dataset. The best test power for each dataset is highlighted in bold. The suffix "-q" denotes MMD-quad and "-l" MMD-lin. SA: *Staphylococcus aureus*, EC: *Escherichia coli*

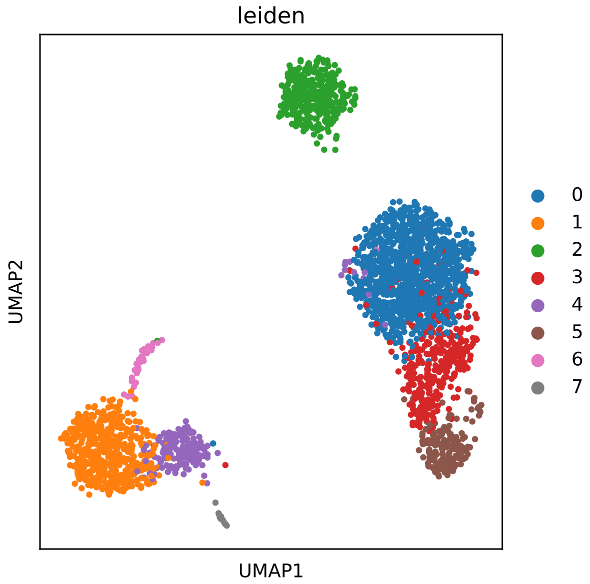

Figure S 9: A u-map representation of the PBMC gene expression dataset colored by cluster membership.

Table 4: Logistic regression binary classification performance based on different experimental data sets with different feature selection approaches as indicated based on the same number of features as identified by SpIn-Opt-l. univar.FS: feature selection by univariate association, (significant): using all significant features, (fixed #): using a fixed number of features equal to the number of significant MMD-l features, -: no significant features found.

E Hyperparameter Sweep

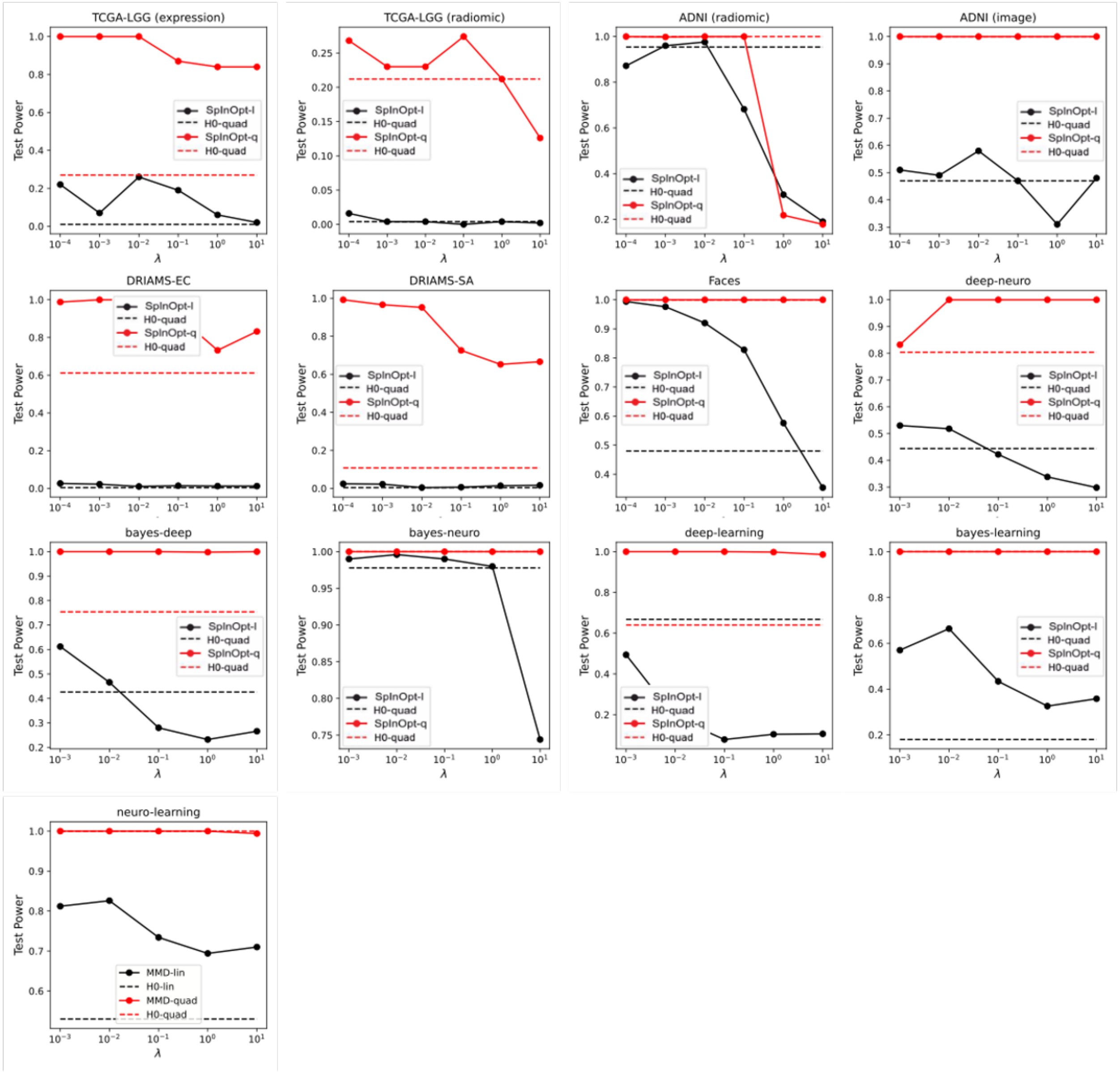

Figure S 10: Test power as a function of lambda for the experimental results.

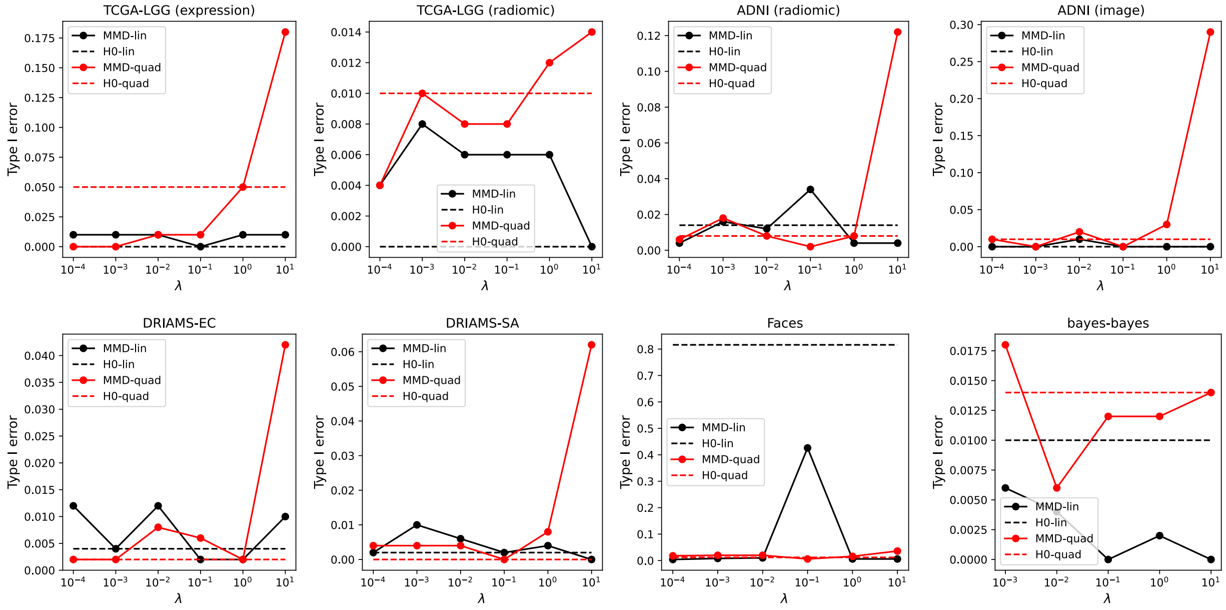

Figure S 11: Type I error as a function of lambda for the experimental results.

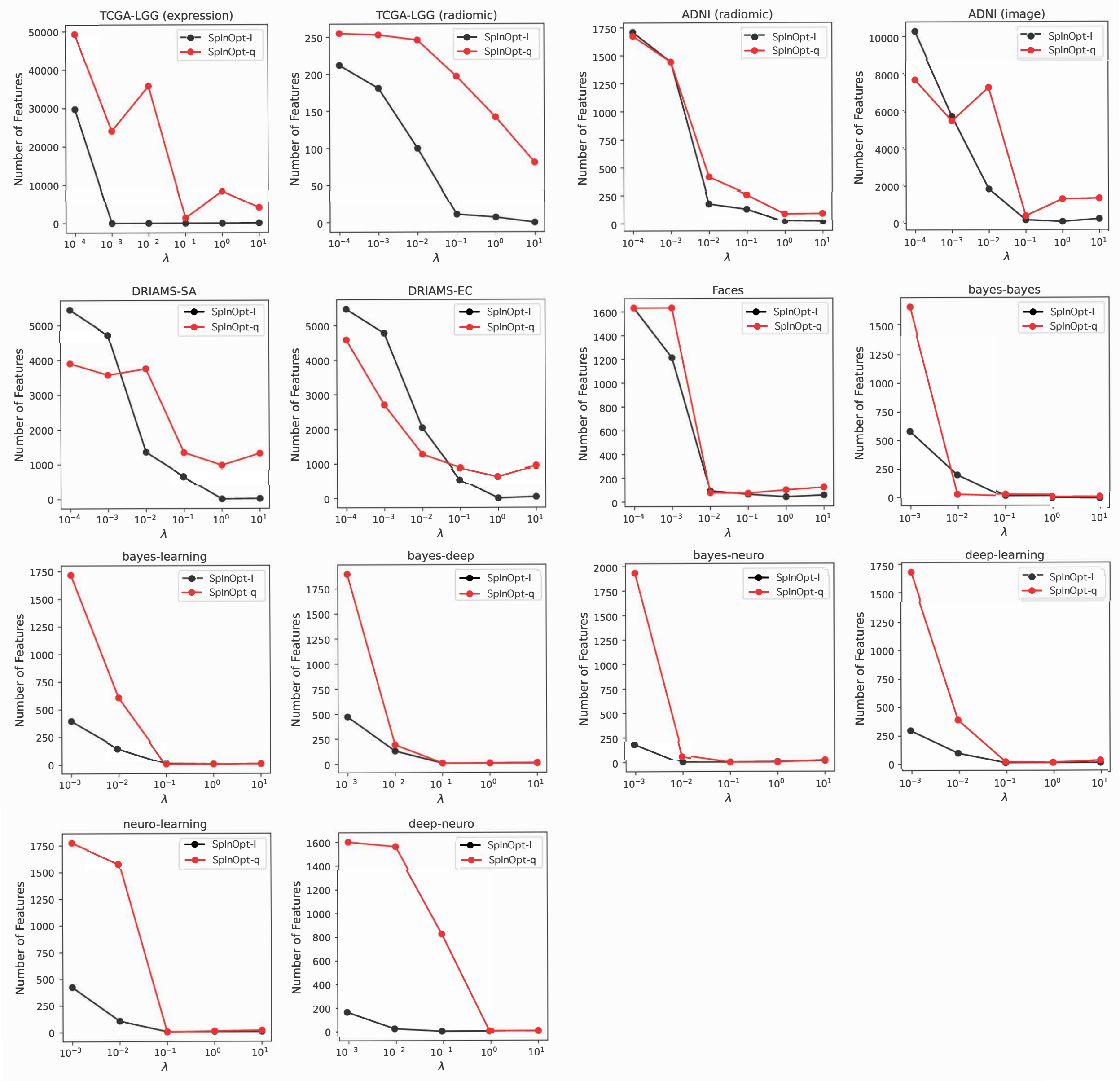

Figure S 12: Number of selected features as a function of lambda for the experimental results.

F Addtional Acknowledgements

Data collection and sharing for this project was funded by the Alzheimer's Disease Neuroimaging Initiative (ADNI) (National Institutes of Health Grant U01 AG024904) and DOD ADNI (Department of Defense award number W81XWH-12-2-0012). ADNI is funded by the National Institute on Aging, the National Institute of Biomedical Imaging and Bioengineering, and through generous contributions from the following: AbbVie, Alzheimer's Association; Alzheimer's Drug Discovery Foundation; Araclon Biotech; BioClinica, Inc.; Biogen; Bristol-Myers Squibb Company; CereSpir, Inc.; Cogstate; Eisai Inc.; Elan Pharmaceuticals, Inc.; Eli Lilly and Company; EuroImmun; F. Hoffmann-La Roche Ltd and its affiliated company Genentech, Inc.; Fujirebio; GE Healthcare; IXICO Ltd.;Janssen Alzheimer Immunotherapy Research & Development, LLC.; Johnson & Johnson Pharmaceutical Research & Development LLC.; Lumosity; Lundbeck; Merck & Co., Inc.;Meso Scale Diagnostics, LLC.; NeuroRx Research; Neurotrack Technologies; Novartis Pharmaceuticals Corporation; Pfizer Inc.; Piramal Imaging; Servier; Takeda Pharmaceutical Company; and Transition Therapeutics. The Canadian Institutes of Health Research is providing funds to support ADNI clinical sites in Canada. Private sector contributions are facilitated by the Foundation for the National Institutes of Health (www.fnih.org). The grantee organization is the Northern California Institute for Research and Education, and the study is coordinated by the Alzheimer's Therapeutic Research Institute at the University of Southern California. ADNI data are disseminated by the Laboratory for Neuro Imaging at the University of Southern California.

References

[1] Kacper P Chwialkowski, Aaditya Ramdas, Dino Sejdinovic, and Arthur Gretton. Fast two-sample testing with analytic representations of probability measures. *Advances in Neural Information Processing Systems*, 28, 2015.

[2] Arthur Gretton, Dino Sejdinovic, Heiko Strathmann, Sivaraman Balakrishnan, Massimiliano Pontil, Kenji Fukumizu, and Bharath K Sriperumbudur. Optimal kernel choice for large-scale two-sample tests. *Advances in neural information processing systems*, 25, 2012.

[3] Wittawat Jitkrittum, Zoltán Szabó, Kacper P Chwialkowski, and Arthur Gretton. Interpretable distribution features with maximum testing power. *Advances in Neural Information Processing Systems*, 29, 2016.

[4] Daniel Lundqvist, Anders Flykt, and Arne Öhman. Karolinska directed emotional faces. *Cognition and Emotion*, 1998.
